# Diagnostic pattern of mental, neurological and substance use disorders at primary health care facilities in Uganda

**DOI:** 10.1186/s13033-024-00643-9

**Published:** 2024-07-15

**Authors:** Byamah B. Mutamba, Gad Twikirize, Jimmy Ssemalulu, Roseline Babirye, Lynn Semakula, David Cappo

**Affiliations:** 1 YouBelong Uganda, Kampala, Uganda; 2 Butabika National Referral Mental Hospital, Kampala, Uganda

**Keywords:** Mental, Neurological, Substance use, Primary health care, Utilization, Health services, Health information

## Abstract

Integration of diagnosis and treatment for mental, neurological, and substance use (MNS) disorders into primary health care is a recommended strategy to improve access to services in low-and middle-income countries. Despite numerous initiatives for integration of mental health care in Uganda, there has not been an evaluation of health management information system (HMIS) records to determine whether MNS disorders are routinely diagnosed. We sought to determine diagnostic pattern of MNS disorders at primary health facilities in Wakiso and Kampala districts, the most populous regions of Uganda. Lower-level primary health facilities were visited to obtain records from HMIS registers, to document diagnoses of MNS disorders. Secondary data analysis was conducted and descriptive statistics reported. A total of 40 primary health care facilities were visited representing 58.6% of the health facilities in the study districts. More than half (54.8%) and almost all (87.5%) of the lower-level health facilities in Wakiso district and Kampala district respectively were visited. The proportion of MNS disorders diagnosed at lower-level primary health facilities in Uganda is very low with Epilepsy the most common MNS diagnosis recorded. Reasons for such low numbers of diagnoses at primary health facilities are discussed as are possible solutions.

## Background

The mental health treatment gap is largest in low resourced settings where up to 90% do not receive evidence-based care [1, 2]. Many barriers to treatment exist in these contexts, particularly stigma and discrimination, and a poorly resourced mental health service where in most cases a lack of trained health workers results in inadequate mental health care provision [1].

Uganda is a low-income country and in 2016 had a population of 34.6 million people with a 3% projected annual population growth rate [3].The treatment gap for MNS disorders in Uganda is estimated to be as large as 85%^4^. The national health policy prescribes integration of mental health into primary health care as a strategy to improve access to mental health services (Kigozi,2007) (National Health Policy 2010). Integrating mental health care into routine primary health care can optimize both mental health and physical health outcomes and avoid fragmentation of health services (Collins et al., 2013, Patel et al., 2013). Theoretically, integration can be effectively done within general health care facilities by non-specialist health providers that are provided brief training and appropriate supervision by mental health specialists (Eaton et al., 2011, World Health Organization, 2010). Whether this has translated into accessible and adequate mental health care services in Uganda is subject to further study, but current evidence from health management information system (HMIS) records suggests otherwise [4]. The one national mental hospital in Uganda, Butabika National Referral Mental Hospital, a tertiary component of the mental health system continues to attend to the bulk of mental, neurological and substance use disorder (MNS) cases as the ‘first port of call’ and not the primary health system [5].

However, there is limited knowledge about the state of MNS services in lower level public health facilities also known as health centres (Health Centre (HC) II- IV) in Wakiso and Kampala districts. These facilities, which are within the catchment area of Butabika National Referral Mental Hospital. are required to provide MNS services.

YouBelong Uganda (YBU), a non-governmental organization (NGO) registered in Uganda, envisions and works toward the development of a long-term sustainable system of mental health care in the country, that is available and accessible to the population [6–8]. As part of its strategic plan to help develop and strengthen key components of an emerging mental health system in Uganda, YBU initiated a study to determine the pattern of MNS diagnoses at lower public health facilities (HC II, III and IV) in Wakiso and Kampala districts of Uganda [9]. We sought to determine the pattern of MNS diagnoses recorded at lower- level public health facilities.

## Methods

**Fig. 1:**
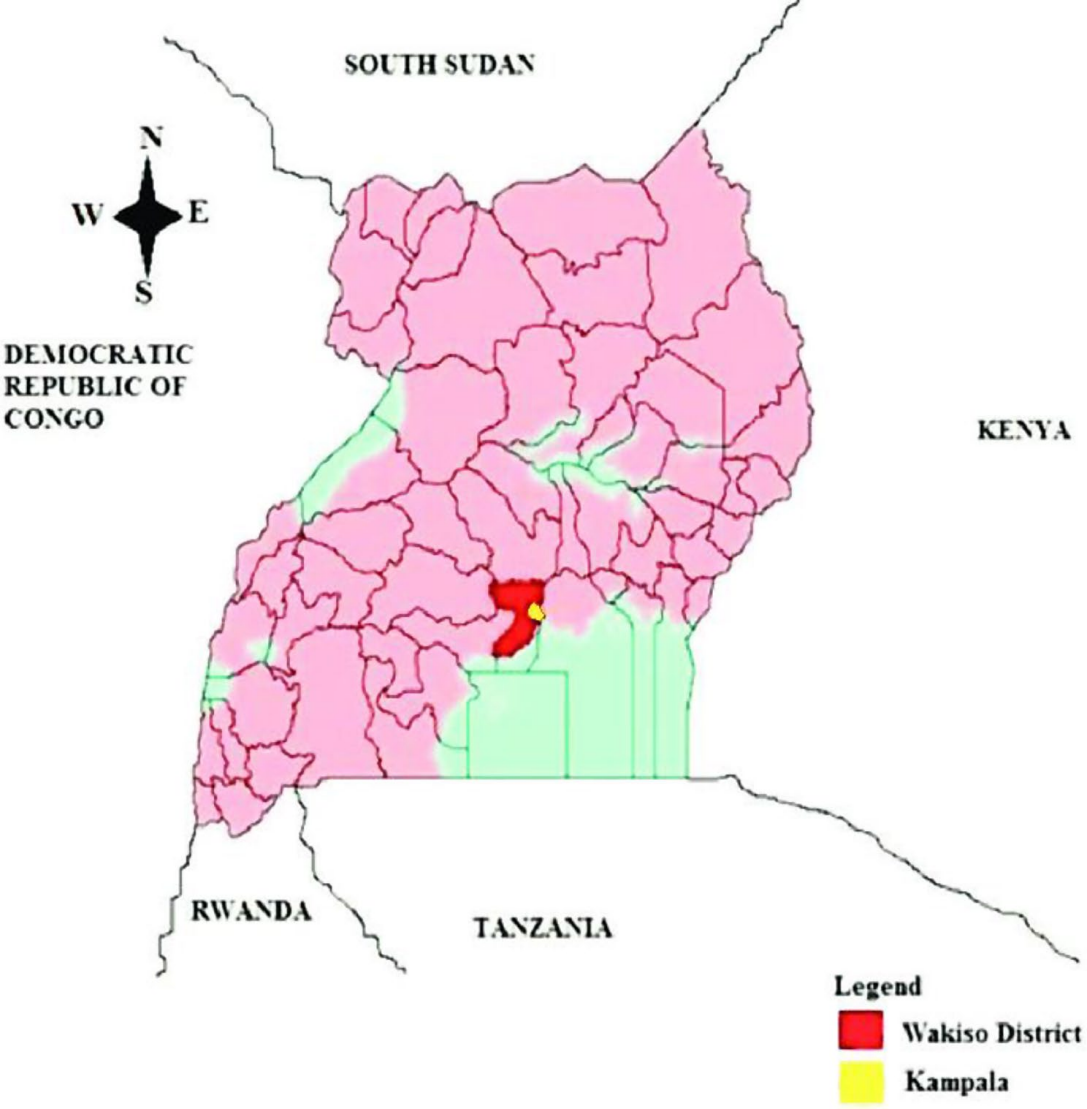
Map of Uganda showing Kampala and Wakiso districts

The study was carried out from August 2019 to January 2020 in the districts of Wakiso and Kampala which are located in the central region of Uganda (see Fig. 1). The two urban and peri urban districts of Kampala and Wakiso are located in central Uganda, with a total population of 1,507,080 and 1,997,418 respectively.

Kampala District is divided into five divisions including Kawempe, Makindye, Central, Rubaga and Nakawa (Table 1).Wakiso District is divided into two counties of Busiro and Kyadondo. Busiro County has the following health sub districts: Busiro South, Entebbe municipality, Busiro North and Busiro East. Kyadondo County has the health sub districts of Kyadondo South, Kyadondo North, Kyadondo East and Kira.

**Table 1:** Population of the administrative divisions of kampala

Division	Male population	Female population	Total
Central	37,435	37,733	75,168
Kawempe	158,768	179,897	338,665
Lubaga	176,762	206,454	383,215
Makindye	186,368	206,640	393,008
Nakawa	153,429	163,594	317,023
Total	712,762	794,318	1,507,080

The Uganda primary health care system is organized according to the different levels of health facilities; from the lowest to the highest level. At the lowest level (the village) are community health workers (CHWs), who are mainly tasked with health promotion activities, a level two (II) health centre serves a parish and provides general outpatient health services, a level three (III) health centre serves a sub county and provides inpatient maternal health care in addition to general outpatient services, and the next level is health centre four (IV) which serves a district population and provides non-specialist inpatient and outpatient care.Different cadres of primary health care workers (PHWs) staff the health facilities; the number and range of cadres is dependent on the level of health facility. Health centre IV staff include Medical Officers (MOs), Medical Clinical Officers (MCOs), General nurses, and midwives. There may also be specialist mental health workers including Psychiatric Clinical Officers (PCOs) and mental health nurses at this level. Health centre III staff include MCOs, general nurses, midwives and mental health nurses. HC IIs are staffed by general nurses only [10].

Health facilities were purposefully selected based on prior engagement with YouBelong Uganda including those whose mental health and/or other health worker staff had been trained by YouBelong Uganda in 2018. Selected health facilities included seven of the 8 lower-level public health facilities (HC II-HC IV) in Kampala district and 33 of the 62 lower public health facilities in Wakiso district.

A survey methodology was employed using quantitative approaches: a document review of facility reports including the Health Management Information System (HMIS)105 form was conducted, after which it was manually extracted and entered into a prepared data sheet premised on the HMIS 105. The team reviewed the Health Management Information System (HMIS) 105 section on MNS diagnoses for the financial year 2018/19; a period of 12 months. The HMIS form 105 is a summation form for monthly cases reviewed at a health facility [11]. At the time of the study, it had a section on mental health including bipolar disorder, depression, epilepsy, dementia, childhood mental disorders, schizophrenia, HIV related psychosis, anxiety disorder, alcohol abuse, drug abuse and a category listed as ‘other mental health disorders. After January 2020, the HMIS 105 form was revised to include more MNS conditions.

A statistician entered the HMIS data in excel and conducted descriptive analyses. Frequencies were generated and illustrated using tables and graphs. Because of the limitations of secondary data, analyses to determine statistically significant associations were not done.

Statistical tests of significance can provide valuable insights into relationships and associations within the data, but it is essential to recognize the constraints imposed by the nature of secondary data we used and therefore we had to exercise caution on drawing conclusions or making inferences beyond the scope of the descriptive analyses that we conducted. We used preexisting data routinely collected by health workers for HMIS, a purpose unrelated to our specific research objectives. In addition, the data did not align perfectly with our variables of interest. This limited our ability to conduct statistical significance tests,

The other limitation was the collection constraint; we had no control over the data collection process because we relied on data collected by others and not ourselves, which may introduce bias, inconsistences or missing information. Furthermore, the secondary data used lacked detailed contextual information about sample characteristics at individual level or potential confounding factors which poses a challenge in assessing the validity of assumptions underlying statistical tests.

Hence, the secondary data available to us could not provide sufficient statistical power or precision to detect meaningful effects or relationships.

Because of these constraints associated with the secondary data utilized, we found it inappropriate and unreliable to conduct statistical significance tests on it. We therefore focused on providing descriptive insights into the dataset, including frequency distributions and visual representations to facilitate understanding and interpretation.

## Results

The study was carried out in lower-level health facilities (HCII- HCIV) in Wakiso and Kampala Districts. A total of 40 health facilities were visited in the two districts representing 58.6% of the health facilities in the study districts. More than half (54.8%) of the lower health facilities in Wakiso district and almost all (87.5%) of the lower-level health facilities in Kampala district were visited Table 2.

## Kampala district

Seven (of eight) lower-level health facilities in Kampala were visited by the YBU study team, including 5 health centre IIIs (Komamboga health centre III in Kawempe division, Kisuggu health centre III in Makindye division, Kawaala and Kitebi health centre IIIs in Rubaga division, Kiswa health centre III), one health centre II (Bukoto health centre II in Nakawa division), and one health centre IV (Kisenyi health centre IV in Central division).

### Proportion of MNS diagnoses at primary health care facilities

Mental, neurological and substance use (MNS) disorders contributed only 1.25% of the total health related diagnoses recorded at all the lower health facilities in Kampala District. Mental Disorders, Epilepsy and Substance Use Disorders contributed 0.58%, 0.59% and 0.08% of the total diagnoses respectively, indicating very low service delivery in primary care for MNS disorders in comparison to services for other health conditions.

**Fig. 2:**
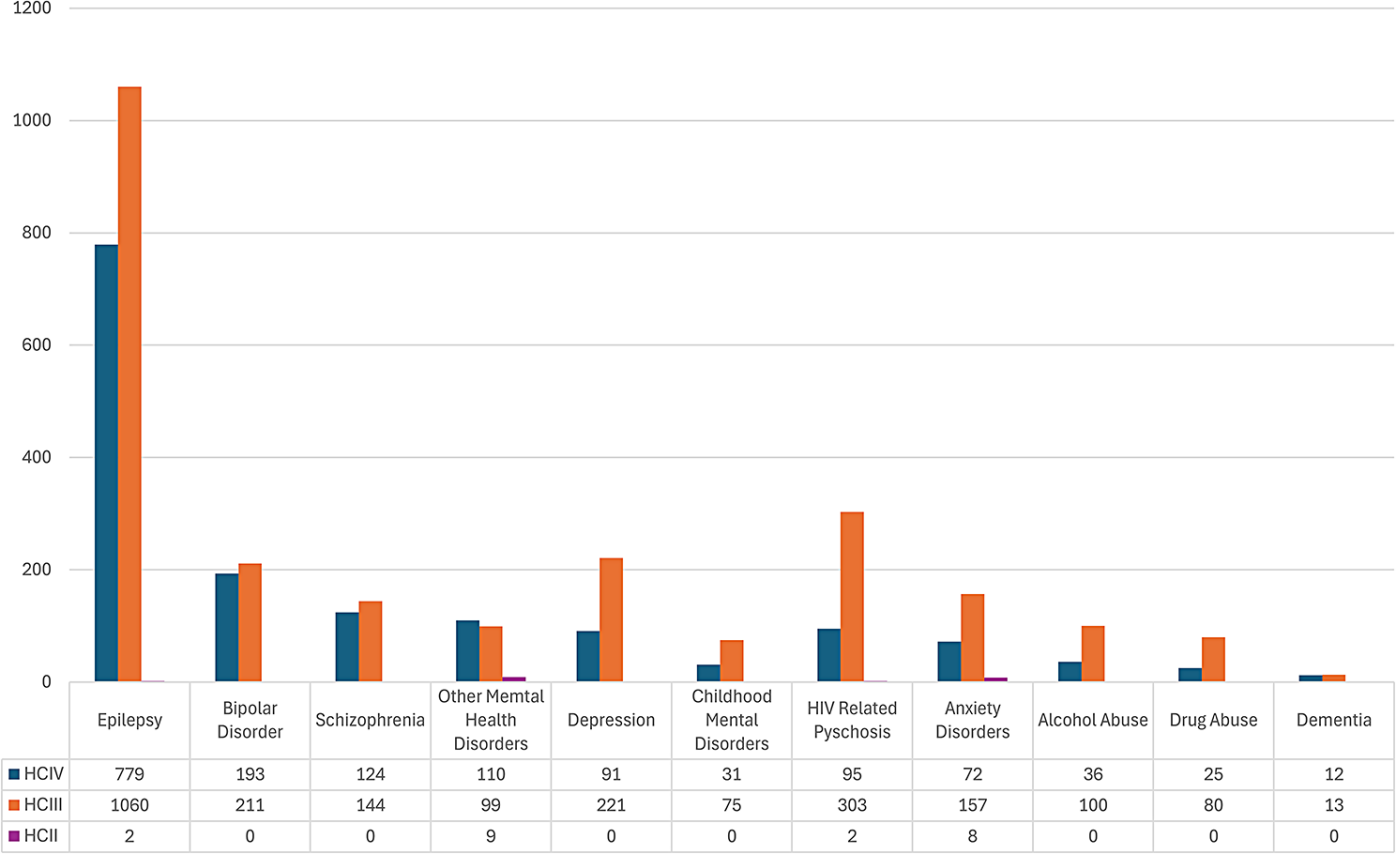
Number and proportion of MNS cases diagnosed at facilities in Kampala district

As shown in Fig. 2 and Table 2, Epilepsy (45%) was the most common MNS disorder recorded at health facilities, particularly at HC III and IV. Bipolar disorder (10%), HIV related psychosis (10%) and Depression (8%) were the other commonly diagnosed mental disorders. Of note is the low proportion of alcohol and substance use disorders, both at 3%, recorded at these levels. Level II health facilities recorded only 4 diagnostic categories: HIV Psychosis, Anxiety, Epilepsy and Other Mental Disorders. Other Mental Disorders (43%) was the most frequent diagnosis recorded at level II facilities, followed by Anxiety at 38%. By far, most diagnoses of MNS disorders were recorded at HC III; Epilepsy accounted for almost half (43%) and half (50%) of MNS disorders recorded at level III facilities and at level IV facilities respectively, in Kampala district. Whereas HIV Related Psychosis (12%) was the second most common diagnosis recorded at HCIII, Bipolar disorder (12%) was the second most common diagnosis recorded at HCIV.

**Table 2:** Proportion and number of MNS disorders recorded at lower-level health facilities in Kampala district

Diagnosis	HCIV	%HCIV	HCIII	%HCIII	HCII	%HCII	Total	%Total
Epilepsy	779	50%	1060	43%	2	10%	1841	45%
Bipolar disorder	193	12%	211	9%	0	0%	404	10%
Schizophrenia	124	8%	144	6%	0	0%	268	7%
Other mental health disorders	110	7%	99	4%	9	43%	218	5%
HIV related psychosis	95	6%	303	12%	2	10%	400	10%
Depression	91	6%	221	9%	0	0%	312	8%
Child hood mental disorders	31	2%	75	3%	0	0%	106	3%
Anxiety disorders	72	5%	157	6%	8	38%	237	6%
Alcohol abuse	36	2%	105	4%	0	0%	141	3%
Drug abuse	25	2%	75	3%	0	0%	100	2%
Dementia	12	1%	13	1%	0	0%	25	1%
Total mns cases	**1568**	**100%**	**2463**	**100%**	**21**	**100%**	**4052**	**100%**

**Fig. 3:**
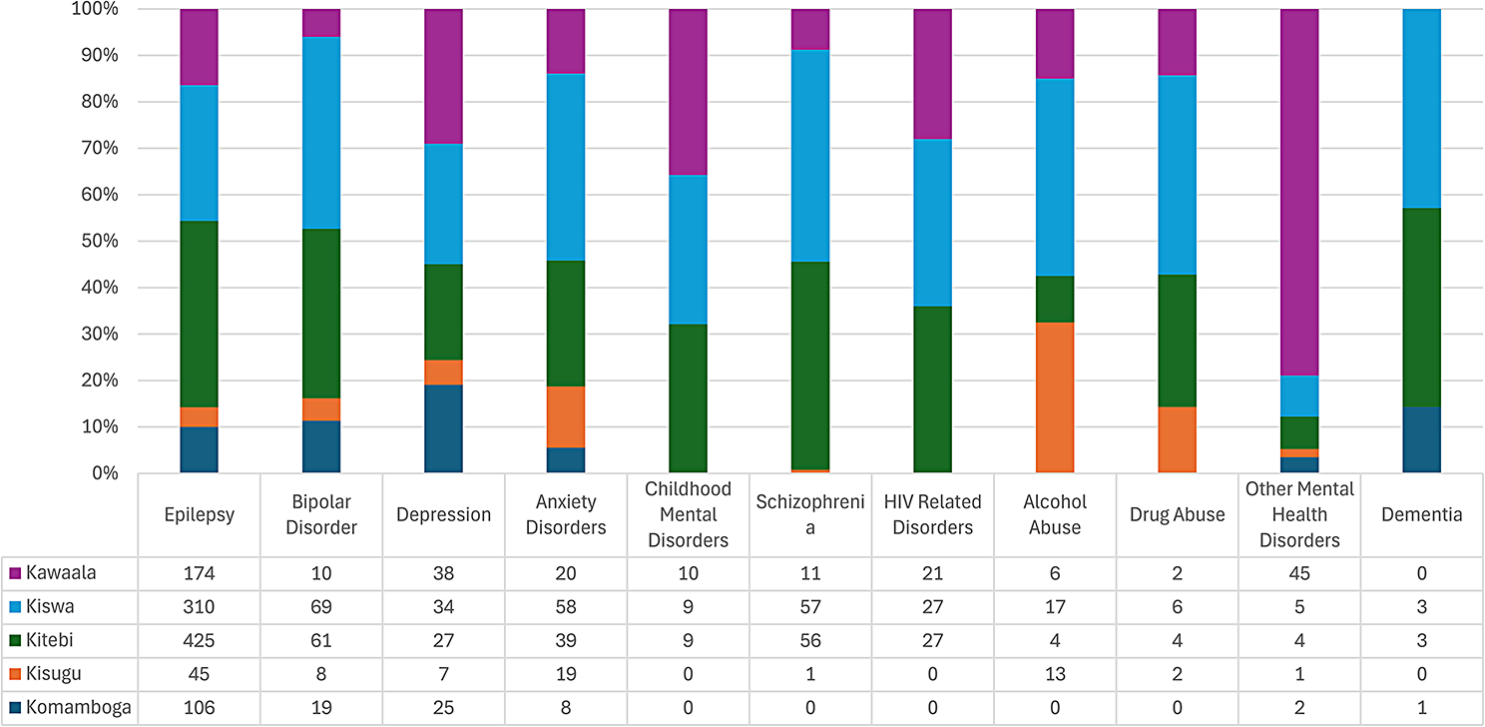
Diagnoses of MNS disorders at HC III in Kampala district

At level III health facilities, Epilepsy (45.1%) was the most commonly diagnosed MNS disorder followed by HIV related psychosis (11.5%) and Depression (8.5%). All MNS diagnostic categories were recorded at all level III facilities except for Drug and Alcohol abuse at Komamboga, HIV related psychosis at Kisuggu and Komamboga, Schizophrenia at Komamboga, Childhood Mental Disorders at Kisuggu and Komamboga, and Dementia at Kisuggu and Kawaala (Fig. 3).

**Fig. 4:**
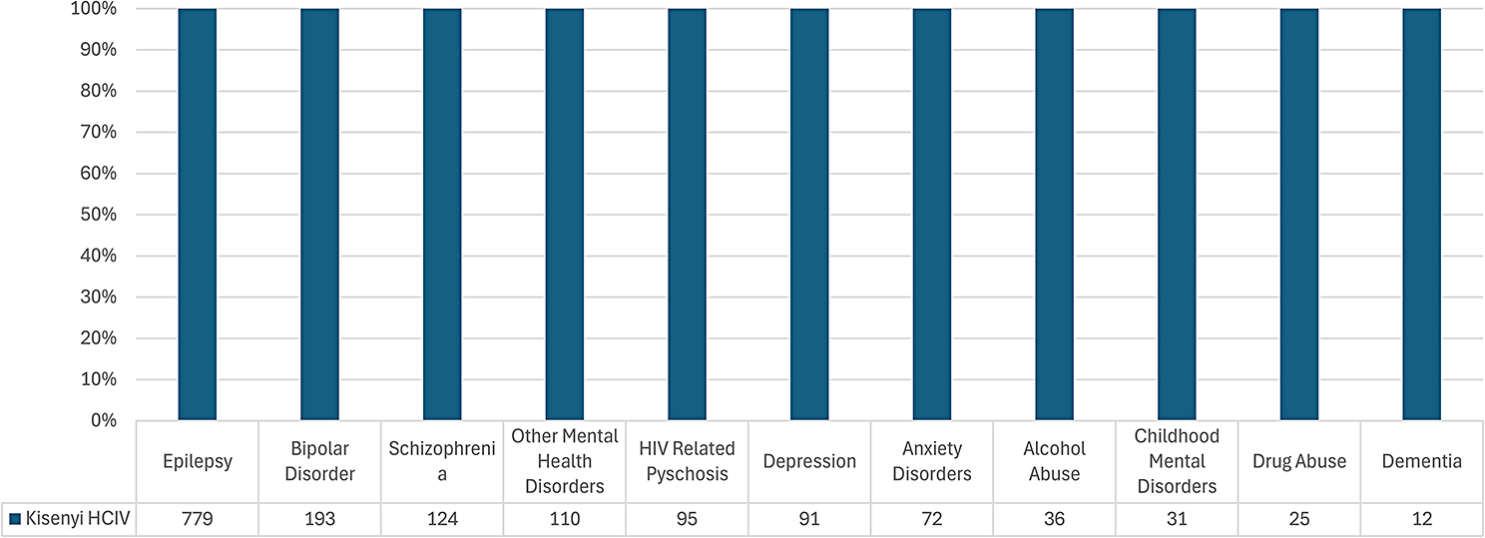
Diagnoses of MNS disorders at HC IV in kampala district

Recorded attendance at the only level IV facility visited (Kisenyi HCIV) was highest for Epilepsy, Bipolar disorder, Schizophrenia and other mental disorders In descending order (Fig. 4).

### Mental health personnel at health facilities in Kampala district

Of the 7 health facilities visited in Kampala district, only Kisenyi health centre IV had a trained and qualified mental health worker as part of assigned personnel (Table 3).

**Table 3:** Availability of mental health personnel by level of health care facility in Kampala district

	HC II	HC III	HC IV
Cadre			
Enrolled psychiatric nurse	0	0	1
Psychiatric nursing officer	0	0	0
Psychiatric Clinical officer	0	0	0
Total	0	0	1

### WAKISO DISTRICT, UGANDA.

A total of 33 health facilities were visited in Wakiso district. These included six health centre IVs, seventeen health centre IIIs and ten health centre IIs. These facilities were located in the health sub districts of Busiro East, Busiro North, Busiro South, Entebbe Municipality, Kyadondo North, Kyadondo East and Kyadondo South.

**Fig. 5:**
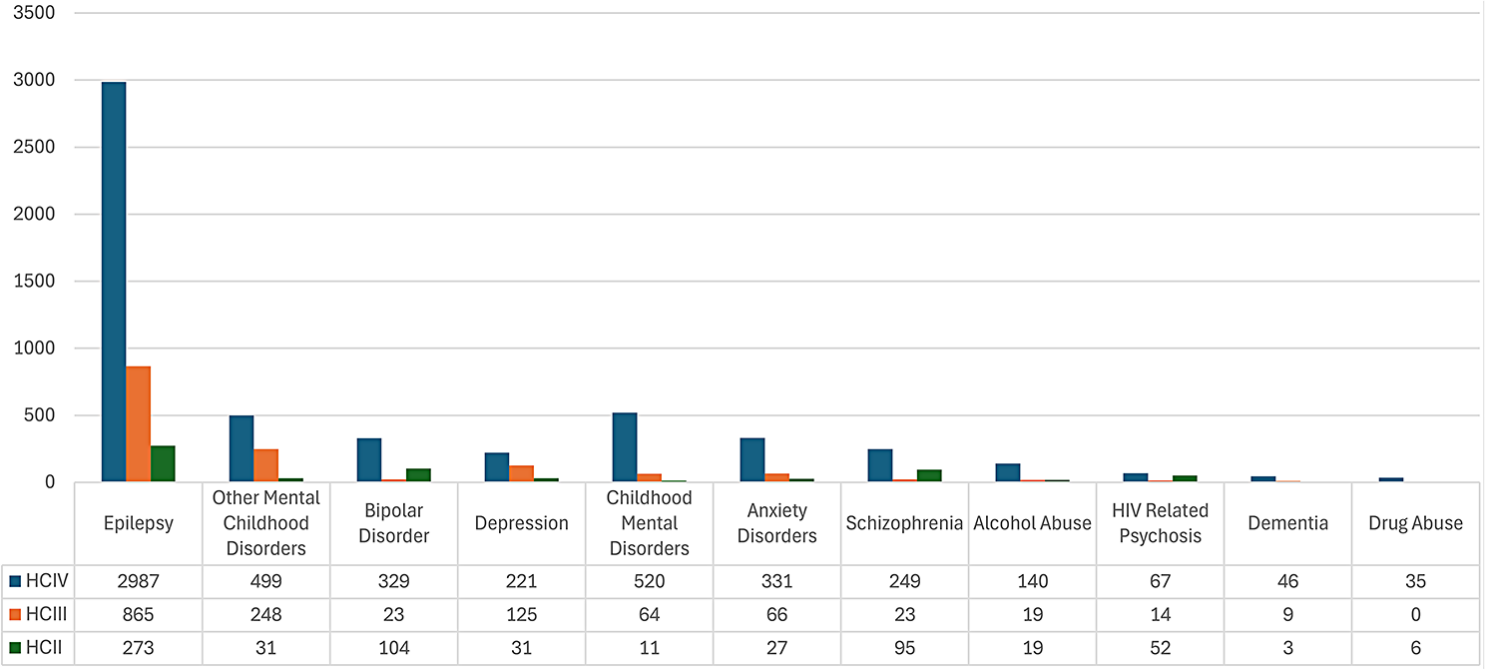
Proportion of MNS disorders recorded at different levels of health facilities in Wakiso district

MNS disorders accounted for 1.76% of the total diagnoses recorded at the 33 lower-level health facilities visited in Wakiso district. Epilepsy was by far the most common MNS disorder recorded at all levels of health facilities in Wakiso district (Fig. 5). The MNS disorders frequently recorded at all HCs visited in Wakiso district included: Other Mental Health Disorders (14.3%), Bipolar disorder (7.7%), Childhood Mental Health Disorders (6.8%) and Schizophrenia (6.1%). Bipolar Affective disorder and Schizophrenia were diagnosed in equal proportions at HC IV and II; both these diagnoses and that of Depression, Childhood mental disorders, HIV Psychosis and Drug abuse were recorded more frequently at HC II than HC 111.

Table 4 shows that Epilepsy (55%) was the most common diagnosis recorded at all levels of health facilities. At health centre II Bipolar (16%), and Schizophrenia (15%) were the second and third most commonly recorded diagnoses. At health centre III, it was Other Mental disorders (17%), and Depression (9%), and at health centre IV, it was Childhood Mental disorders (10.1%) and Other Mental Disorders (9%) and.

It is also of note that, apart from Epilepsy, Dementia, Anxiety and Other Mental disorders, health centre IIs recorded more diagnoses of MNS disorders, particularly for Severe Mental Illness (Bipolar, Schizophrenia, Depression, HIV Psychosis), compared to health centre IIIs.

**Table 4:** Proportion and number of mns disorders recorded at lower-level health facilities in wakiso district

Diagnosis	HCIV	%HCIV	HCIII	%HCIII	HCII	%HCII	Total	%Total
Epilepsy	**2987**	**55%**	**865**	**59%**	**273**	**42%**	**4125**	**55%**
Other mental health disorders	**499**	**9%**	**248**	**17%**	**31**	**5%**	**728**	**10%**
Bipolar disorder	**329**	**6%**	**23**	**2%**	**104**	**16%**	**459**	**6%**
Depression	**221**	**4%**	**125**	**9%**	**31**	**5%**	**337**	**4%**
Child hood mental disorders	**520**	**10%**	**64**	**4%**	**11**	**2%**	**595**	**8%**
Anxiety disorders	**331**	**6%**	**66**	**5%**	**27**	**4%**	**421**	**6%**
Schizophrenia	**249**	**5%**	**23**	**2%**	**95**	**15%**	**367**	**5%**
Alcohol abuse	**140**	**3%**	**19**	**1%**	**19**	**3%**	**178**	**2%**
HIV related psychosis	**67**	**1%**	**14**	**1%**	**52**	**8%**	**133**	**2%**
Dementia	**46**	**1%**	**9**	**1%**	**3**	**0%**	**58**	**1%**
Drug abuse	**35**	**1%**	**0**	**0%**	**6**	**1%**	**41**	**1%**
Total mns cases	**5424**	**100%**	**1456**	**100%**	**652**	**100%**	**7532**	**100%**

**Fig. 6:**
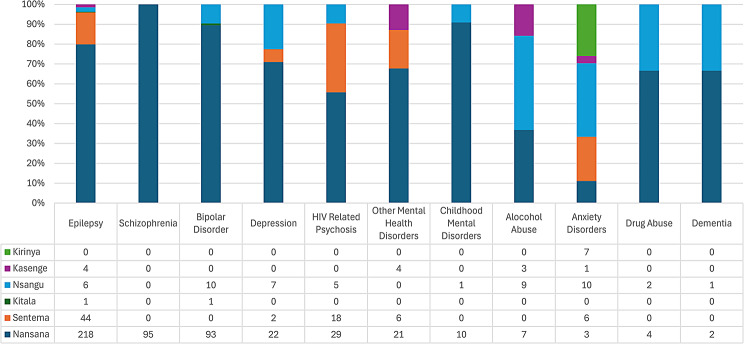
Diagnoses of MNS disorders at health centre IIs in Wakiso district

Excepting Nansana HC II, there was evident low number of diagnoses recorded at level II health facilities in Wakiso district. Figure 6 shows that Nansana health centre II stands out as the facility with the highest number of diagnoses recorded for all MNS disorders compared to the other health centre IIs. The majority of the Wakiso district health centre IIs did not register a diagnosis of mental and substance use disorders. One facility (Kitala) registered only one diagnosis and two health facilities (Nakitokolo and Matuga) did not record any diagnosis of over the 12-month period. Epilepsy was the most frequent diagnosis recorded at this level.

**Fig. 7:**
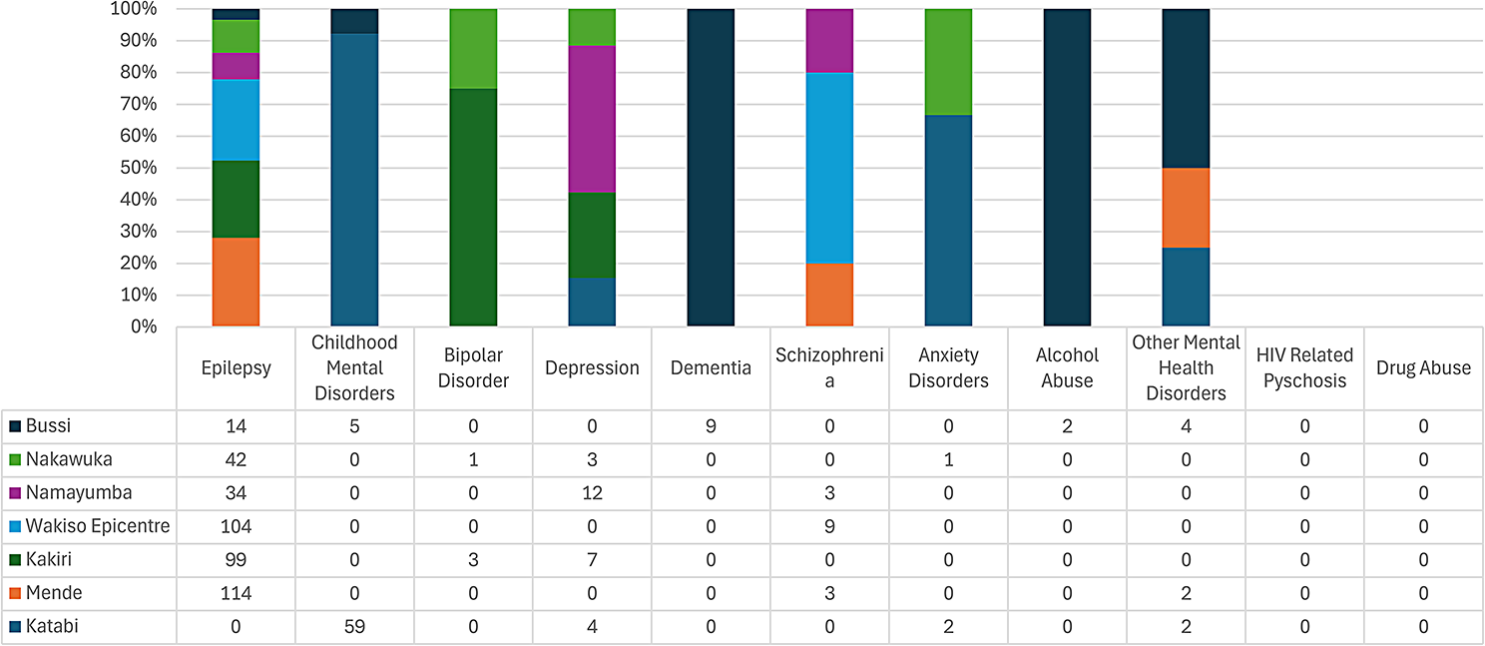
Diagnosis of MNS disorders recorded at various health centre IIIs in Wakiso district

**Fig. 8:**
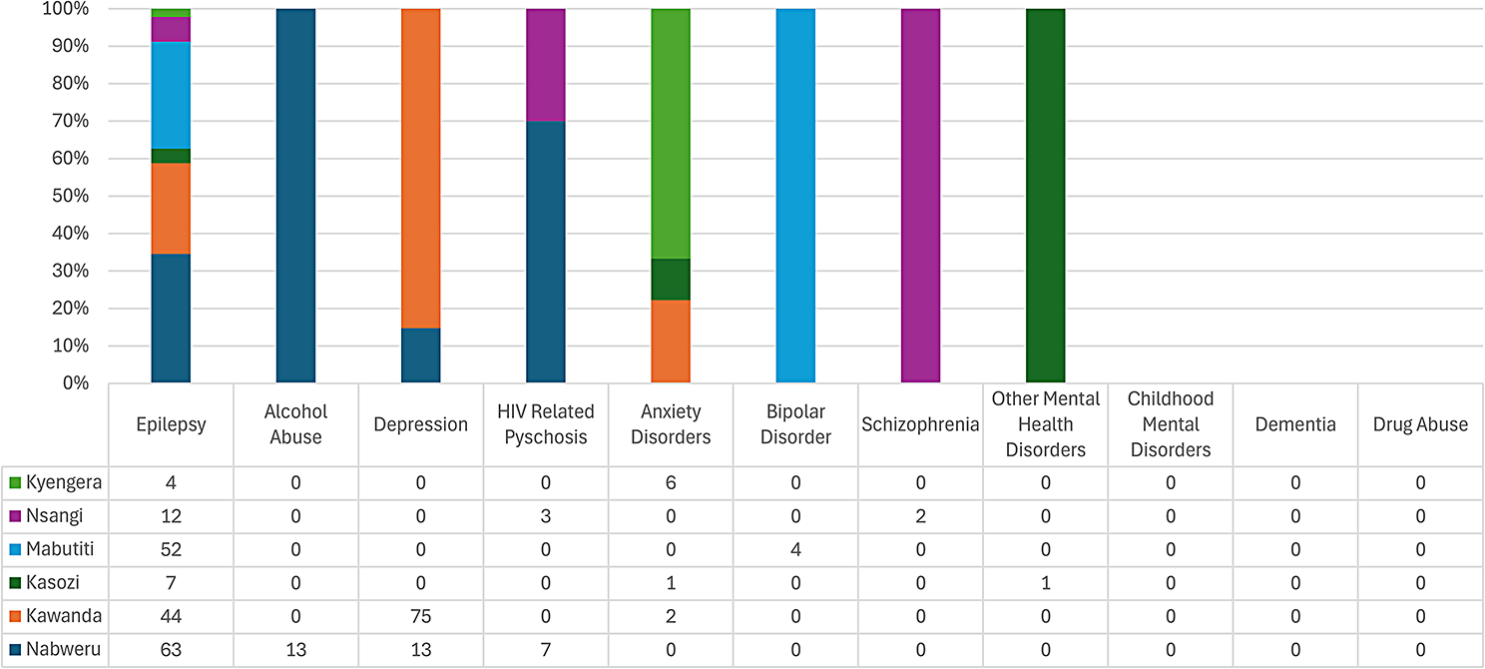
Diagnosis of MNS disorders recorded at various health centre IIIs in Wakiso district

**Fig. 9:**
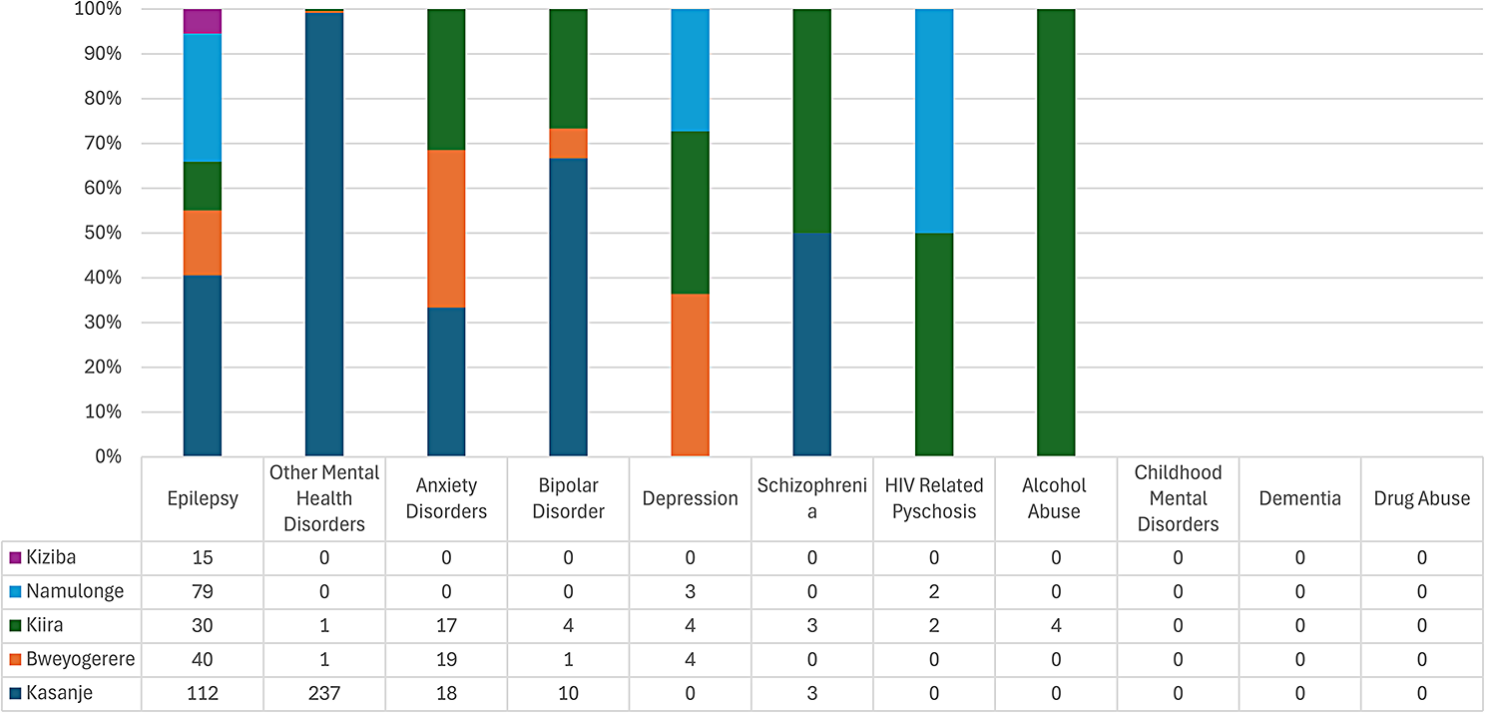
Diagnosis of MNS disorders recorded at various health centre IIIs in Wakiso district

With respect to level III health facilities in Wakiso district, Epilepsy was the most common reason for attendance at all health centres (Figs. 7, 8 and 9), however, there were peculiarities at particular facilities: Nabweru HC III recorded comparatively higher number of diagnoses for Alcohol and HIV Psychosis, as did Kawanda HC III for Depression, Wakiso HC III for Schizophrenia, Katabi HC III for Dementia and Bussi HCIII for Childhood Mental Disorders (the only level III health facility in Wakiso District with a recorded diagnosis for Childhood Mental Disorders).

The high numbers of ‘Other Mental Health Disorders’ and Anxiety diagnoses recordedat Kasanje HCIII is notable.

In general, there was a very low numbers of MNS disorders recordedat HCIIIs in Wakiso district during the study period.

**Fig. 10:**
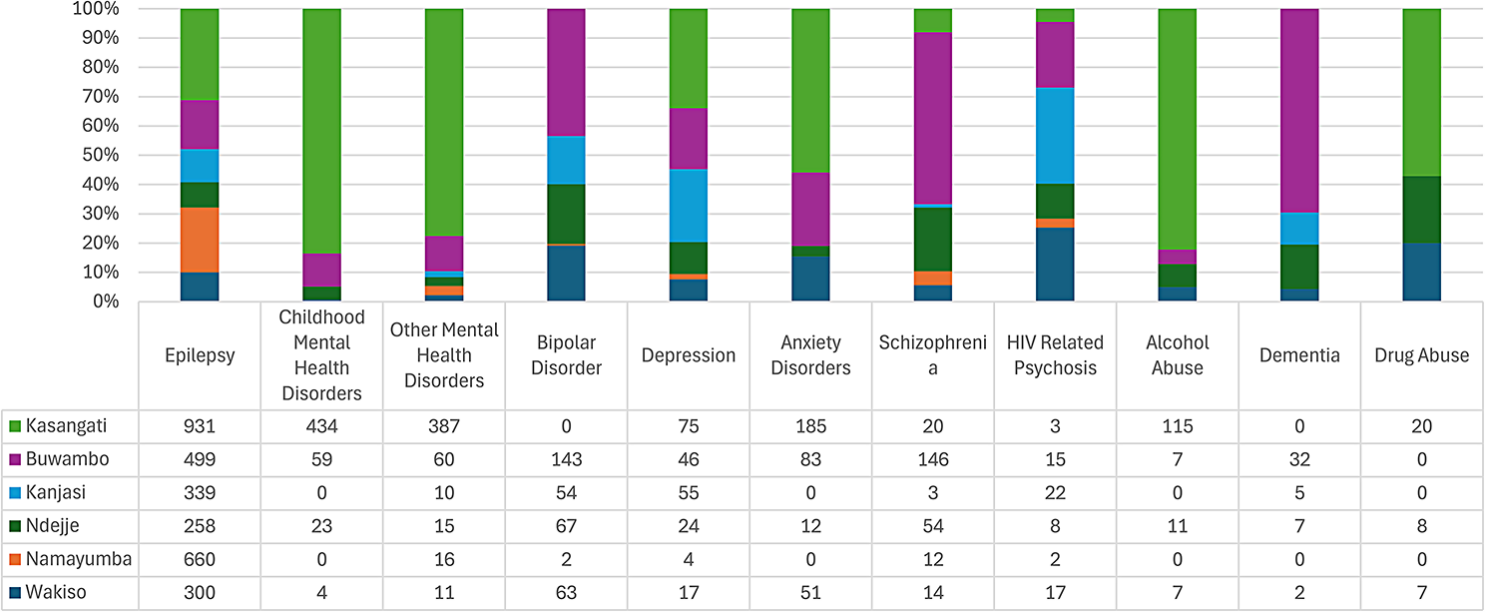
Diagnosed of mental, neurological, substance use disorders recorded at health centre IVs in Wakiso

Records of MNS disorders diagnosed at level IV health facilities in Wakiso were similar to the trend at level II and III facilities (Fig. 10): Epilepsy (52.7%) was the most frequent diagnosis, with much lower frequencies for mental and substance use disorders. Other mental disorders (10.4%) and childhood mental disorders (10.1%) were the 2nd and 3rd most common category of diagnoses respectively. Notably, Kasangati and Buwambo health centres had comparatively higher numbers recorded for a range of MNS disorders. Also of note, is that Kasangati HCIV recorded a comparatively higher number of diagnoses overall than other HCIVs, followed by Buwambo and Ndejje HCIVs.

#### Mental health personnel at health centres in Wakiso district

Only two health facilities (Kasangati health centre IV and Kira health centre III) had Psychiatric clinical officers. The other health centre IVs had enrolled psychiatric nurses. The other health centre IIIs and IIs did not have a psychiatric nurse deployed at the facilities (Table 5).

**Table 5:** Availability of mental health personnel by level of health care facility in Wakiso district

	HC II	HC III	HC IV
Cadre			
Enrolled psychiatric nurse	0	0	3
Psychiatric nursing officer	0	0	0
Psychiatric Clinical officer	0	1	1
Total	0	1	4

## Discussion

The study investigated the pattern of diagnosis for MNS disorders using the HMIS platform at lower-level public health facilities in two districts of Uganda.

Study findings show that MNS cases accounted for less than 2% of the total diagnoses recorded at lower-level health facilities in both study districts. The zero cases of Severe Mental Illness (Bipolar, Schizophrenia, Depression), Dementia, and Alcohol/Drug use disorders at level II facilities in Kampala District, with Other Mental Disorders being the largest category recorded (Fig. 2), may imply the lack of competency to diagnose MNS disorders at this level. This is consistent with findings from a study by Ae-Ngibise and others, 2023 who found a low detection rate of people with MNS conditions in routine primary healthcare facilities. Up to 98% of MNS conditions were not detected [12].

Hence, most of the MNS disorders diagnosed at the facility are ‘lumped’ into ‘Other Mental Health Conditions’: a diagnostic category in the Uganda Health Management Information system under which conditions that do not fit the diagnostic criteria of Bipolar, Depression, Epilepsy, Dementia, Childhood mental disorders, Schizophrenia, HIV Related Psychosis, Anxiety Disorders, Alcohol and Drug use disorders are meant to be recorded.

In Wakiso District, the picture was similar except for Nansana HC II where the number of MNS disorders diagnosed was comparatively high: It is worth noting that Nansana HC II runs a monthly mental health outreach clinic supported by specialist mental health workers from Butabika mental hospital, which could explain the high numbers.

The high proportion of cases diagnosed as Anxiety may also lend more credence to the ‘lumping’ phenomenon, considering that Depression, which frequently lies on the continuum of Aanxiety and other common mental disorders [13] did not register even one diagnosis at any of the health facilities in Kampala. That said, the relatively high number of diagnoses due to HIV related psychosis raises even more questions about diagnostic competencies, as to how health workers who are able to diagnose psychosis related to HIV, are not able to diagnose psychosis of Bipolar or Schizophrenia. The evidence suggests that non specialist health workers are more inclined to the biomedical model of (mental) illness, where all illnesses have a specific cause or are secondary to a medical cause (e.g., secondary psychosis), rather than the biopsychosocial model of causality. Roberts and others found that primary health workers were more inclined to diagnose physical rather than psychological symptoms [14]. Another reason could be that there is more training of health workers in HIV management than there is for other conditions. Semakula et al.,2020 found that health workers tend to think biomedical for most diagnoses and do not take into consideration the psychological and social aspects of (mental) illness [15].

Recording of MNS disorders improved at level III health facilities in Kampala District but not in Wakiso District (where the majority of facilities did not record a single case of MNS disorder). This could be explained by the availability of specialist mental health workers at some of the facilities in Kampala district compared to those in Wakiso. The specialist mental health workers support delivery of MNS services in the health facilities. It is also worth noting that Kitebi health center III conducts a monthly mental health outreach clinic supported by Butabika mental hospital as does Kiswa health center which runs a child and adolescent clinic that includes ‘psychosocial/mental health’ services. However, overall, the cases recorded at level III facilities highlight a critical gap in primary health services: the lack of capabilities to diagnose MNS disorders. It is expected that level III HCs which have a larger human resource, and should have a better diagnostic capability than level II. This discrepancy warrants further study.

The level IV facilities in Kampala District indicated higher numbers of diagnoses for severe mental illness (SMI) including Bipolar disorder and Schizophrenia, but there was a mixed picture for level IV facilities in Wakiso District which had higher attendances for both SMI and ‘Other Mental Health disorders’. The higher recorded numbers for SMI would suggest high levels of diagnostic competencies compared to lower-level health facilities. As exemplified by Kasangati HC IV, there were high numbers of childhood mental disorders and alcohol abuse diagnoses (which may indicate high diagnostic capabilities), however, there were also high numbers for ‘Other mental health disorders’ and anxiety, suggesting lack of diagnostic comptencies among the same health workers.

This paradox at level IV facilities may be due to a number of reasons: 1) Some of the level IV health facilities, albeit a few, have specialist mental health workers including Psychiatric Clinical Officers (PCOs) and Psychiatric nurses as part of their staff, which should increase the competencies to correctly diagnose MNS disorders. This may explain the high number of MNS cases recorded at Kasangati HC IV and Buwambo HC IV which facilities have PCOs on the staff but also brings into question why there is no single case of Bipolar recorded at Kasangati. Other possible explanations include diagnostic misclassification, the staff have more diagnostic skills, the centres receive a higher number of referred cases from lower-level facilities or the facilities have more MNS medicines available in stock that cater to the need of persons previously diagnosed with SMI and attending the facility for medicine refills.

2) Level IV facilities have a wide catchment area (see Fig. 1) and are referral units for lower level (II and III) facilities hence higher numbers of MNS cases would be expected at this level. However, this probably holds true if reason 1 above exists and may explain the low numbers recorded at Wakiso, Namayumba and Kajjansi HC IV which do not have specialist mental health workers on their staff.

Study findings from Wakiso district indicate a diagnostic pattern where lower-level health facilities, with less qualified staff, are able to diagnose more MNS disorders than those at a higher level, which have more and higher qualified staff. The zero record of mental and substance use disorders diagnosed at the majority of level III health facilities over a 12 months period is another notable finding. The presence of trained personnel who have a particular focus on one MNS category e.g., childhood mental disorders, and on the other hand the limited or lack of mental health training of general health workers would be factors of influence in the above observations.

The high number of 503 Other Mental Health Disorders at Kasanje III demands further inquiry as is the finding that Kira HC IV that has a PCO, does not show high numbers of MNS disorders recorded. The comparatively high number of MNS diagnoses recorded at Kasangati for Childhood Mental Disorders is of particular note, and analysis of this factor could point to further development of what constitutes a service that would attract childhood specific mental health service attendees.

On the whole, for both Kampala and Wakiso districts, the low number of diagnoses recorded for MNS disorders at level II, III and IV facilities (in comparison to the total attendance of all conditions recorded) could be because persons with MNS disorders or their family members seek care from higher level facilities or alternative care practitioners (traditional and faith healers). Turiho and others (2018) found that the majority of persons with SMI and their families did not consider lower-level health centers/facilities as their first port of access despite them being near and accessible; the reasons advanced included cultural/traditional causes of the illness and a perceived lack of competency of health workers at this level to manage mental health problems [7, 16].

The proportion of MNS disorders diagnosed at lower level health facilities is very low compared to other illnesses considering that national prevalence estimates indicate that over 35% of the general population in Uganda are affected [17]. The number of substance use disorder cases was relatively low compared to neurological and mental disorders. This could be due to the low awareness among the communities about the availability of services to treat substance use disorders at lower health facilities. Those who would have benefited from the services at the lower public health facilities may also have poor health seeking behaviour. As hypothesized above for mental disorders, a lack of knowledge and skills in the management of substance use disorders among primary health care workers could also be a factor in under/misdiagnosis of these health conditions [12, 18].

Further, beyond the level of health worker training and skills, more questions arise about access to care, and stigma towards MNS disorders held by health workers, patients and their families [14]. It is difficult to fathom that majority of level III health facilities in Wakiso, each serving a population of at least 20,000 people, did not register a case of MNS disorder yet level II facilities were able to. Turiho and others (2018) found that a significant number of patients and their carers affected by Severe mental illness and Epilepsy bypassed the lower-level facilities because of a perceived lack of care and competence at these levels [16]. Evidence from elsewhere has shown that stigma by health workers is a major factor in access to services for MNS disorders [19–21]. This has been complicated by a lack of diagnostic skills among general health workers to manage mental health disorders at primary health centres [12, 19].Stigma and lack of awareness in local communities has been cited as one of the barriers to access and utilization of mental health services at the lower level public health facilities [10, 20, 21].

Epilepsy was the most common MNS diagnosis recorded at all health facilities in Kampala and Wakiso districts. This is consistent with findings by Molodynski and others (2017) who observed that more people suffer from Epilepsy than mental disorders in Uganda [17]. It may also be an indication of the limited capacity at this level of the health system to diagnose mental disorders i.e., the mental treatment gap. Because, in as much as Epilepsy was the most common diagnosis, recorded numbers are still lower than expected considering evidence of prevalence and the treatment gap (TG) for persons living with epilepsy (PLWE) [22, 23], and the likelihood that health workers at this level lack the skills to diagnose non-convulsive Epilepsy [9, 19].

In low resourced settings like Uganda, Epilepsy is considered to be a mental disorder as much as it is a neurological disorder. The general population perceive it as a ‘brain disease’ hence the reason it frequently presents to mental health services. Kaddumakusa and others (2015) in their study of community members in Uganda found that 39.8% and 30.2% of the study participants thought that Epilepsy was a neurological and mental illness respectively [24]. Because of this and other reasons, it is classified under mental health conditions [9, 20]. The behavioural and cognitive symptoms of both convulsive and non-convulsive epilepsy, and the stigma associated with Epilepsy are similar to that of persons living with mental illness [25]. That mental health services are perceived as more understanding and accepting of patients with Epilepsy are some of the other reasons that may explain the conceptualization of Epilepsy as a mental disorder. Epilepsy contributes significantly to the global disease burden but the majority of those affected, about 80%, are resident in low- and middle-income countries (LMIC) [26] with most of these countries located in Africa [23].

Irrespective of the cultural, social, economic and geographic context, there are common factors underlying the treatment gap for Epilepsy: poor health system infrastructure including a lack of trained health care personnel and inadequate supplies of antiepileptic drugs, cultural beliefs, stigma and unavailability of Anti Epilepsy Drugs (AEDs) [22, 27, 28].

The evidence above on the epilepsy TG is consistent with findings from the YBU Baseline study which shows that limited service options, cost and affordability of care, lack of transport, the distances people have to travel for mental health care and a perception that there is a lack of mental health care in the non-specialty health facilities because “health workers at lower level health facilities lack the confidence to diagnose and treat people with mental illness”, were the barriers to care for persons living with Severe Mental disorders and Epilepsy, and their families [16].

The epilepsy treatment gap in LMICs highlights the need to not only integrate epilepsy treatment into existing general/primary health care services considering the inadequate numbers of neurologists and/or mental health workers [9, 10, 21], but also to incorporate knowledge and skills to manage psychosocial aspects of epilepsy [9, 22, 29, 30].

Our findings are limited by the use of secondary data which was not comprehensive enough to conduct extensive data analysis. However, in future where primary data collection is undertaken or access to comprehensive datasets is available, we may explore the possibility of conducting statistical significance tests to further discover relationships and associations of interest. In addition, we used total numbers of MNS diagnoses and considering that some patients e.g. those living with Epilepsy are return patients who attend the facility recurrently, this may inflate the number of diagnoses recorded. Another limitation of the study is that the diagnoses were made by non-specialists which may bring into question the reliability and validity of some of the diagnoses, but this was the aim of the study: to reveal the reality of MNS diagnostic competencies and related services at this level of the health system.

## Conclusion

Perhaps the most significant finding of this study is the extremely low numbers of MNS diagnoses at lower level primary health facilities in the Uganda public health system within a context of the unmet burden of MNS disorders in Uganda.

Reasons for such low attendance at lower level primary health facilities could be attributed to the following: Stigma and shame attached to MNS disorders with such stigma encompassing a person’s family as well as the person concerned; cultural preference towards using traditional and faith healers; cost of transport in accessing services; public awareness that medications are more likely to be available at tertiary level hospitals than primary care services; knowledge of lack of availability of mental health support and the lack of trained mental health practitioners at most lower level health facilities; and very few general primary health workers with mental health training.

Whilst this study only focused on two geographic districts, there is nothing to suggest that the same pattern of low numbers of MNS disorders would not pertain to other districts throughout Uganda. Dedicated training programs that take into consideration the local health system context can significantly improve the competencies of non-specialist primary health workers in diagnosis and treatment of MNS disorders. A health facility, alongside classroom-based didactic training program, to improve health worker competencies, is one for future consideration. Utilising the role of community health workers as a referral function of locally identified people and their families affected by MNS disorders, to health centres, is a further initiative to be considered.

## Data Availability

The datasets used and/or analysed during the current study are available from the corresponding author on reasonable request. All data generated or analysed during this study are included in this published article.

## Abbreviations

AED: Anti Epilepsy Drugs
HC: Health Centre
HIV: Human Immunodeficiency Virus
HMIS: Health management information system
LMIC: Low- and Middle-Income countries
MNS: Mental, Neurological, and substance use disorders
NGO: Non-governmental Organisation
QOL: Quality of Life
PCO: Psychiatric Clinical Officer
TG: Treatment Gap
YBU: YouBelong Uganda
